# Functional and Nonclinical Similarity of ABP 980, a Biosimilar of Trastuzumab

**DOI:** 10.1007/s11095-019-2702-8

**Published:** 2019-11-06

**Authors:** Shea Jassem, Wei Wang, Heather Sweet, Raffi Manoukian, Vincent Chow, Palanisamy Kanakaraj, Katariina M. Hutterer, Scott Kuhns, Ian N. Foltz, Qing Chen, John Ferbas, Helen J. McBride

**Affiliations:** Amgen Inc, One Amgen Center Drive, Thousand Oaks, California 91320 USA

**Keywords:** ABP 980, biosimilar, breast cancer, gastric cancer, trastuzumab

## Abstract

**Purpose:**

The *in vitro* and *in vivo* pharmacologic assessment of ABP 980 similarity to its reference product is intended to compare the activity of ABP 980 and trastuzumab and support the overall conclusion of similarity based on a comprehensive analytical and functional evaluation.

**Methods:**

This work complements the primary assessment of functional similarity with additional *in vitro* assays, binding studies, and non-clinical studies including human epidermal growth factor receptor-2 (HER2) kinetic binding, HER2 signaling, HER2 internalization, synergy with docetaxel chemotherapy, FcγR kinetic binding, primary natural killer and monocyte cell binding, antibody-dependent cellular phagocytosis activity, *in vivo* xenograft studies, and toxicokinetic parameters.

**Results:**

The results contribute to the totality of evidence with respect to functional similarity and support that ABP 980 is similar to trastuzumab in all primary and secondary mechanisms of action.

**Conclusions:**

These results also support the scientific justification of extrapolation to all approved indications of trastuzumab given the established functional similarity of the two products and the same mechanisms of action across all conditions of use.

**Electronic supplementary material:**

The online version of this article (10.1007/s11095-019-2702-8) contains supplementary material, which is available to authorized users.

## Introduction

ABP 980 (KANJINTI™) was developed as a biosimilar to trastuzumab (HERCEPTIN®, Genentech Inc.). KANJINTI™ was approved by the EMA on May 16, 2018 and by the FDA on June 13, 2019 for the same indications approved for Herceptin including human epidermal growth factor receptor-2 (HER2)-overexpressing early breast cancer, metastatic breast cancer, and metastatic gastric cancer. Trastuzumab is a recombinant immunoglobulin G1 (IgG1) monoclonal antibody that binds HER2 and is approved for the treatment of metastatic breast cancer, early breast cancer, and metastatic gastric cancer (1,2). Human epidermal growth factor receptors (HERs) are a family of 4 transmembrane tyrosine kinase receptors that mediate cell growth, differentiation, and survival (3). HER2 (also referred to as Neu, ErbB-2) is 1 member of this family of proteins. Over-expression of HER2 has been shown to correlate with aggressive tumors (4,5). Treatment of cancer cells over-expressing HER2 with trastuzumab, a therapeutic antibody targeting HER2, results in inhibition of HER2-mediated mitogenic signaling and a reduction in cell proliferation (6).

A biosimilar is a biologic drug that is similar to an approved, branded biological reference product (7–11). Reference products and intended biosimilars share the same amino acid sequence, but as opposed to generics, are not expected to be identical due to the complexities associated with biologics. Minor structural and functional differences are unavoidable due to expression systems that are often proprietary. Differences may also exist in cell culture, purification techniques, and formulation between a biosimilar and its reference product, and more so between biosimilars of the same reference product. These minor differences are acceptable as long as they do not impact efficacy, safety, purity, or potency of the proposed biosimilar. Overall, the demonstration of similarity is based on a totality of evidence (TOE) approach and requires a step-wise comparison that begins with analytical evaluation, which encompasses structural, functional, and physiochemical comparisons (12,13). Depending on the results of the initial similarity assessment, non-clinical studies including pharmacology and toxicology studies may be conducted to reduce residual uncertainty around the biosimilar product, although such studies, particularly those conducted in non-human primates, are generally not recommended (8).

Demonstration of similarity in clinical pharmacokinetics, and if available, pharmacodynamics, is also expected as part of the TOE approach; this is followed by a comparative clinical evaluation of efficacy, safety, and immunogenicity in a sensitive population at the same approved dose and route of administration as the reference product in a representative indication using sensitive endpoints. When all the results are combined together, the biosimilarity of a molecule is evaluated in the whole.

The evalution of residual uncertainty and the need for additional testing to resolve it for biosimilar development has evolved over time, particularly as more comparisons of clinical efficacy and safety have become available, demonstrating no clinically meaningful differences between biosimilars and their reference products even with minor analytical differences reported (14), While the utility of nonclinical *in vivo* studies is recommended to be more targeted to address specific aspects of residual uncertainty, the breadth of *in vitro* functional assessments used to evaluate similarity has increased to ensure any potential impact on all reported functions of a molecule have been thoroughly evaluated (11,15).

As part of the foundation for the TOE to support the similarity of ABP 980 to trastuzumab, a comprehensive analytical and functional similarity assessment demonstrated that ABP 980 is highly similar to trastuzumab with some minor analytical differences observed (16). The pharmacokinetic and clinical studies supporting the similarity of ABP 980 to trastuzumab have also been published (17–19). The studies presented here complement the comprehensive functional assessment with additional *in vitro* binding (HER2 relative cell binding and binding kinetics, FcγR cell and kinetic binding), additional aspects of effector and primary HER2 inhibition (ADCP, inhibition of HER2 signaling, inhibition of proliferation in gastric cancer cells, synergy with chemotherapeutic and HER2 internalization) as well as non-clinical pharmacology (tumor xenograft studies in breast and gastric cancer models) and toxicokinetic results. These results provide additional confidence in the similarity of ABP 980 and trastuzumab for all functional aspects of the molecules *in vitro* and *in vivo* and contributed to the initial TOE supporting the determination of biosimilarity of ABP 980 and the scientific justification of extrapolation of indications.

## Materials and Methods

For each set of data described in this section, replicates and any statistical methods employed are defined. All qualitative studies are representative of at least 2 replicates.

### HER2 Binding Kinetics

The kinetics of binding to rHER2 (Amgen Inc.) were determined by SPR using a ProteOn XPR36 optical biosensor (Bio-Rad, Hercules, CA, USA) and a general layer compact sensor chip (Bio-Rad, Hercules, CA, USA) with single cycle kinetics. Samples were captured to the ProteOn chip surface using a goat-anti-human IgG1 antibody (Jackson ImmunoResearch Laboratories, Inc., West Grove, PA, USA). The dissociation equilibrium binding constant (Kd) for ABP 980 and trastuzumab binding to rHER2 (amino acids 23–653) were compared. Kinetic rate constants were determined from binding analysis experiments. Five concentrations of rHER2 (analyte) ranging between 25.0 and 0.309 nM were run against captured anti-HER2 antibody on a general layer compact surface. To assess reproducibility of binding and manage potential systematic bias, each of 5 sample concentrations was injected simultaneously for a total of 6 replicates. Blank (buffer) injections were run simultaneously with the 5 analyte concentrations and used to assess and subtract system artifacts. The data were aligned and double referenced using the ProteOn Manager 3.1.0 version 3.1.06 software (Bio-Rad, Hercules, CA, USA). The data were then fit using Scrubber v2.0^©^ software (BioLogic Software Pty Ltd., Campbell, Australia), which is an SPR non-linear least squares regression fitting program. The dissociation rate constant (k_d_) values were determined from fitting the respective 25 nM 3600 s dissociation phase data, and this value was then used as a fixed parameter in the global fits of the 420 s association phase data to a 1:1 binding model to obtain the respective association rate constant (k_a_) values. Equilibrium dissociation constant (K_d_) was then calculated as k_d_ divided by k_a_. Results for ABP 980, trastuzumab (EU), and trastuzumab (US) were reported as the global fits using a 1:1 binding model ± standard deviation of 6 replicates for each lot tested.

### HER2 Cell Binding

A HER2 antigen binding assay was performed using SK-BR-3 cells in a competitive cell-surface binding format. SK-BR-3 cells were assessed for HER2 expression using an Alexa 488-labeled ABP 980 reference standard. A fixed concentration of fluorescently labeled trastuzumab control antibody and increasing concentrations of test articles (ABP 980 or reference material) were incubated with SK-BR-3 cells for 4.5 to 6 h. A dose-dependent decrease in fluorescence signal was detected using an image cytometer (Acumen eX3) with the application of an increasing concentration of unlabeled antibody. The data were fit to a 4-parameter curve and percent relative HER2 binding was reported relative to an ABP 980 reference standard.

### HER2 Signaling: Inhibition of AKT Phosphorylation in BT-474 Cells

Inhibition of AKT phosphorylation was assessed by comparing the effect of test samples on basal pAkt in the HER2-expressing human breast cancer cell line BT-474 (ATCC HTB-20™). Phospho-Akt (Ser473) level was determined using a sandwich immunoassay kit from Meso Scale Discovery (MSD; Rockville, MD, USA). Dilution series of samples and human IgG1 isotype control (Amgen Inc.) were performed in assay media in a 2-fold titration series starting at 10 μg/mL. BT-474 cells (200,000/well) were added to the plates containing titrated antibody before incubating for 4 h. Cells were then lysed, and the cell lysate was transferred to MSD plates pre-coated with anti-Akt capture antibody (MSD; Rockville, MD, USA). After washing, plates were incubated with SULFO-TAG-labeled pAkt (Ser473) detection antibody (MSD: Rockville, MD, USA). A read buffer was added to each well, and the levels of pAkt in the lysate were measured by MSD imager. The percent specific inhibition was calculated as (1 – [signal of treated cells/average signal of untreated cells]) × 100. Dose response curves were plotted using GraphPad Prism^®^ 5.0 software (La Jolla, CA, USA) for visual comparison.

### HER2 Internalization

To assess internalization of target/antibody complex, HER2-expressing SKBR-3 cells were treated with pHrodo™ Red (acidic pH fluorescent indicator) directly conjugated test antibodies for a period of 4 h at 37°C. Control samples were incubated with test antibodies at 4°C to prevent internalization. Two orthogonal approaches were used to observe internalization: flow cytometry, allowing a per cell distribution analysis of pHrodo Red signal, and immunofluorescence confocal microscopy allowing for a qualitative visualization of receptor internalization. Flow cytometric acquisition was performed on an LSRII (Becton Dickinson, San Jose, CA), and analysis was performed using FlowJo flow cytometry analysis software, version 10.4 (Tree Star, Inc., Ashland, OR, USA). Confocal imaging was performed with an Ultraview spinning-disc confocal microscope (PerkinElmer, Shelton, CT, USA) using Volocity image acquisition/analysis software, version 6.3 (PerkinElmer, Shelton, CT, USA).

### Inhibition of Proliferation in NCI-N87 and MCF7 Cells

Inhibition of proliferation of HER over-expressing gastric cancer cells was assessed in NCI-N87 cells (ATCC CRL-5822™), and specificity of inhibition was evaluated using MCF-7 cells (ATCC HTB-22™). Dilution series of samples and huIgG1 isotype control (Amgen Inc.) were performed in assay media in a 2-fold titration series starting at 10 μg/mL and were added to NCI-N87 or MCF7 cells (20,000/well) after plating and allowing time for attachment. Cells were incubated for 3 days, and the cell viability was determined using CellTiter-Glo^®^ (Promega, Madison, WI, USA). Luminescence was measured using a plate reader (EnVision; PerkinElmer, Shelton, CT, USA), and percent inhibition of proliferation was determined using control wells without antibody. Dose response curves were plotted using GraphPad Prism^®^ 5.0 software (La Jolla, CA, USA).

### Synergy Study

All portions of this study were conducted at Horizon Discovery Services, Inc. The method by which Horizon Discovery performed the 2-way synergy assay in NCI-N87 cells has been previously described in detail (20–22). For inhibition of proliferation of NCI-N87 gastric cancer cells (ATCC CRL-5822™), cultures were placed in incubators at 37°C for 24 h before treatment to equilibrate in the assay plates. Assay plates were incubated with test agents for 5 days prior to the addition of ATPLite (Perkin-Elmer, Shelton, CT, USA). Luminescence was measured using an EnVision plate reader, and percent inhibition of proliferation was determined using as a control a duplicate matrix that did not have test agent added. The test agents (4 lots of ABP 980 and 3 lots each of trastuzumab sourced from the US or EU) were profiled in combination with docetaxel (Sigma-Aldrich, St. Louis, MO, USA) using a dose matrix format. Effects on cell proliferation were measured and used to calculate synergy scores for comparison. Additionally, dose response curves were extracted from the matrices and plotted using GraphPad Prism^®^ 5.0 software (La Jolla, CA, USA) for the NCI-N87 synergy studies with and without a single concentration of docetaxel for visual comparison.

The Horizon CombinatoRx™ method was used to examine the synergistic inhibition of proliferation of cancer cells between a second agent (docetaxel) and the test samples (4 lots of ABP 980 and 3 lots each of trastuzumab sourced from the US or EU).

### FcγRIa, FcγRIIa, FcγRIIb, FcγRIIIa, and FcγRIIIb Binding by SPR

Binding was determined in a kinetic binding assay using a Biacore T200 (GE Healthcare, Piscataway, NJ, USA). In brief, His-tagged recombinant human FcγRIa, FcγRIIa (131H), FcγRIIb, FcγRIIIa (158 V), FcγRIIIa (158F), or FcγRIIIb (Amgen, Inc.) was captured by an anti-His antibody (Qiagen, Valencia, CA, USA) on a CM5 Biacore chip (GE Healthcare, Piscataway, NJ, USA). A dilution series of ABP 980, trastuzumab (US), or trastuzumab (EU) was used to measure the binding kinetics in triplicate using a 1:1 kinetic binding model; normalized average sensorgrams from the triplicate data were overlaid for visual comparison using Biacore software (GE Healthcare, Piscataway, NJ, USA).

### Antibody-Dependent Cellular Phagocytosis (ADCP)

Human PBMCs were incubated with CFSE (ThermoFisher, San Diego, CA, USA) labeled SK-BR-3 cells at a 10:1 ratio in an ultra-low adherence 96-well plate. Antibody (samples or isotype control) was serially diluted and added to each well. Cells were subsequently incubated with anti-CD14 antibody (BD Biosciences, San Diego, CA, USA) to stain monocytes and propidium iodide (BD Biosciences, San Diego, CA, USA) to evaluate viability. After washing, 10,000 live CD14+ cells were acquired by FACS. The percent of live CD14+ cells that were positive for CFSE fluorescence was considered to be a direct measure of ADCP activity. Histograms representing the number of events and CFSE fluorescence intensity were plotted and overlaid. Histograms were generated using FlowJo^®^ data analysis software for visual comparison (Ashland, OR, USA).

### FcγR Binding on Primary Monocytes and NK Cells

One donor with a (131 R/R; 158 F/F) genotype and 1 donor with a (131 H/R; 158 V/V) genotype were used for comparative testing. Each test lot was serially diluted and combined with a fixed concentration (200 μg/mL) of Alexa Fluor^®^ 488 fluorescently labeled ABP 980 reference standard. The mixture was then incubated with freshly isolated PBMCs for 1 h, allowing competition for FcγR binding to occur. The samples were then washed, stained with anti-CD56 antibody (BD Biosciences, Franklin Lakes, NJ, USA) and analyzed using a BD FACSCanto II^®^ flow cytometer with BD FACSDiva acquisition software (BD Biosciences, San Diego, CA, USA). The monocyte population was defined by light scatter properties, and the NK cell population was defined by CD56 expression and light scatter properties. When the concentration of unlabeled test lots is low, the Alexa Fluor^®^ 488 fluorescence of the NK and monocyte cell populations is relatively high, due to the low competition for binding of the labeled reference standard. As higher concentrations of unlabeled test lots compete for binding, the fluorescence of the populations decrease. This decrease represents the binding of the unlabeled test lots. For each cell type, histograms representing the frequency of events and Alexa Fluor^®^ 488 fluorescence intensity were plotted. Histograms of test lots at the same concentrations were overlaid and compared visually. Histograms were generated using FlowJo^®^ data analysis software (Ashland, OR, USA).

### Tumor Xenograft Models

Mice were housed at an Association for Assessment and Accreditation of Laboratory Animal Care International (AAALAC)-accredited facility. Animals were cared for in accordance with the Guide for the Care and Use of Laboratory Animals, 8th Edition (23). All research protocols were reviewed and approved by the local Institutional Animal Care and Use Committee.

In the BT-474 (breast tumor) xenograft model, female NOD/SCID mice (China Academy of Medical College Institute of Laboratory Animal Science [CAMCILAS], Beijing, China) at 6 to 8 weeks of age were injected orthotopically with 8 × 10^6^ BT-474 tumor cells per mouse. Animals were group housed and had *ad libitum* access to irradiated, sterilized dry granule food and sterile drinking water. The mice were implanted with estrogen pellets SC to support the growth of BT-474 cells. After 21 days (average tumor size 146 mm^3^), mice were administered vehicle, ABP 980 (0.3 or 3.0 mg/kg), or trastuzumab (EU) (0.3 or 3.0 mg/kg) IV twice weekly for 19 days (*n* = 10 mice/group). Tumor sizes were measured twice weekly until Day 56.

In the NCI-N87 (gastric tumor) xenograft model, immunodeficient female cluster of differentiation (CD)-1 nude mice (Charles River Laboratories CD1-Foxn1^nu^) were injected SC with 5 × 10^6^ NCI-N87 tumor cells per mouse. Animals were group housed and had *ad libitum* access to feed and local tap water (San Diego, CA, USA). When the average tumor size was approximately 100 mm^3^, the mice were administered vehicle, ABP 980 (3 or 10 mg/kg), or trastuzumab (EU) (3 or 10 mg/kg) intraperitoneally twice weekly for 19 days. After treatment was halted, the residual tumors were allowed to regrow, and tumor sizes were measured three times weekly until Day 35.

### Toxicokinetic Analysis

Toxicokinetics of ABP 980 and trastuzumab were evaluated as part of the toxicology study. In this study, 18 female purpose-bred cynomolgus monkeys were assigned to 3 groups (*n* = 6 per group) and dosed with vehicle, trastuzumab (EU), or ABP 980 at 25 mg/kg dose level twice weekly, as IV bolus into the saphenous vein, for 4 weeks. Animals were cared for in accordance with the Guide for the Care and Use of Laboratory Animals, 8th Edition (23). The *in vivo* studies were performed under pre-defined study protocols approved by the Institutional Animal Care and Use Committee. Animals were socially housed at an indoor, AAALAC, internationally accredited facility in species-specific housing. Animals were fed a certified pelleted primate diet (PMI #5048, Richmond, IN) daily in amounts appropriate for the age and size of the animals. Animals had *ad libitum* access to water (municipality tap water processed through a reverse osmosis filter and passed through UV light treatment) via an automatic watering system.

All animals were blood sampled on the first treatment day as well as the last treatment day of the treatment period at pre-dose, 5 min, and 2, 6, 24, 48, and 96 h after dosing. In addition, on the last treatment day, blood sampling also occurred after 168 h after dosing. The serum samples from the blood collections were frozen at −80°C, shipped to a bioanalytical lab (Eurofins Medinet, Breda, The Netherlands), and analyzed to determine the serum concentrations of ABP 980 or trastuzumab. In total, 270 samples were received, and 204 samples were analyzed using a validated colorimetric ELISA method in compliance with the Organisation for Economic Co-operation and Development principles of Good Laboratory Practice (OECD-GLP). The reportable limit of this assay was defined at 200 ng/mL for ABP 980 and trastuzumab with an upper limit of quantification of 10,000 ng/mL. Toxicokinetic parameters were calculated from the individual serum concentrations using WinNonlin v 5.0.1 software (Pharsight Corporation, Mountain View, CA, USA). Toxicokinetic parameters were summarized descriptively. The ratios for AUC values of each treatment group and for each sampling period were calculated and compared to evaluate the time effect and the difference between ABP 980 and trastuzumab.

## Results

### HER2 Binding Kinetics

The biological functions that contribute to the clinical efficacy of trastuzumab are primarily mediated by binding to the extracellular domain of HER2 and subsequent inhibition of downstream ligand-independent signaling events (6). Thus, it is important to confirm that binding kinetics to the HER2 target protein are similar for ABP 980 and trastuzumab. Mature human HER2 consists of 1234 amino acids, in which the N-terminal 631 amino acids are contained within the extracellular domain (ECD), which is subject to cleavage by proteases. Four lots of ABP 980 and 3 lots each of trastuzumab (US) and trastuzumab (EU) were compared for binding to the ECD of HER2 using Surface Plasmon Resonance (SPR). The kinetic data are useful because they provide not only the equilibrium binding constant, but also the rate constants, which characterize the dynamics of the interaction (24). Accurate determination of kinetic binding parameters often requires multiple configurations of test sample and target protein (25). For the purposes of demonstrating similarity, a single method was used here as the purpose is to show that the behavior of the two molecules is similar, not to re-establish the absolute values for target affinity. The results for all tested ABP 980 lots for the parameters of on-rate, off-rate, and relative affinity to HER2 ECD are all contained within the ranges of trastuzumab (EU) and trastuzumab (US), supporting the conclusion that ABP 980 has similar HER2 binding activity (Table I).

**Table I:** Binding Kinetics of ABP 980, Trastuzumab (EU) and Trastuzumab (US) to Recombinant HER2

Sample	k_a_ (M^−1^ s^−1^) × 10^5^	k_d_ (s^−1^) × 10^−5^	K_d_ (pM)
Trastuzumab (EU) Lot #1	6.33	5.77	91.6
Trastuzumab (EU) Lot #2	6.99	4.41	63.0
Trastuzumab (EU) Lot #3	6.67	4.64	69.0
ABP 980 Lot #1	6.64	4.98	75.3
ABP 980 Lot #2	6.61	5.16	78.7
ABP 980 Lot #3	6.54	4.91	74.9
ABP 980 Lot #4	6.65	5.13	76.7
Trastuzumab (US) Lot #1	6.67	5.34	79.5
Trastuzumab (US) Lot #2	7.30	4.90	67.1
Trastuzumab (US) Lot #3	7.24	5.37	74.6

*k*_*a*_ association rate constant, *k*_*d*_ dissociation rate constant, *K*_*d*_ dissociation equilibrium binding constant, *recombinant HER2* human epidermal growth factor receptor 2 extracellular domain, recombinantly expressed, *trastuzumab (EU)* EU authorized trastuzumab, *trastuzumab (US)* FDA licensed trastuzumab, *s* seconds, *M* molar

### HER2 Cell Binding

To evaluate whether ABP 980 has similar cell binding activity as compared with trastuzumab, a competitive cell binding assay using SK-BR-3 breast cancer cells was developed. Four lots of ABP 980 and 3 lots each of trastuzumab (US) and trastuzumab (EU) were compared for evaluation of relative cell binding. The mean relative binding results for all ABP 980 and reference product lots tested were centered around 100% (Table II), supporting the conclusion that ABP 980 and trastuzumab have similar HER2 cell binding activity.

**Table II:** Relative HER2 Cell Binding of ABP 980, Trastuzumab (EU) and Trastuzumab (US)

Sample	Percent Relative HER2 Cell Binding (%)
Trastuzumab (EU) Lot #1	98
Trastuzumab (EU) Lot #2	109
Trastuzumab (EU) Lot #3	108
ABP 980 Lot #1	93
ABP 980 Lot #2	112
ABP 980 Lot #3	104
ABP 980 Lot #4	107
Trastuzumab (US) Lot #1	96
Trastuzumab (US) Lot #2	102
Trastuzumab (US) Lot #3	96

*Trastuzumab (EU)* EU authorized trastuzumab, *trastuzumab (US)* FDA licensed trastuzumab

### HER2 Downstream Signaling through pAKT

**Fig. 1:**
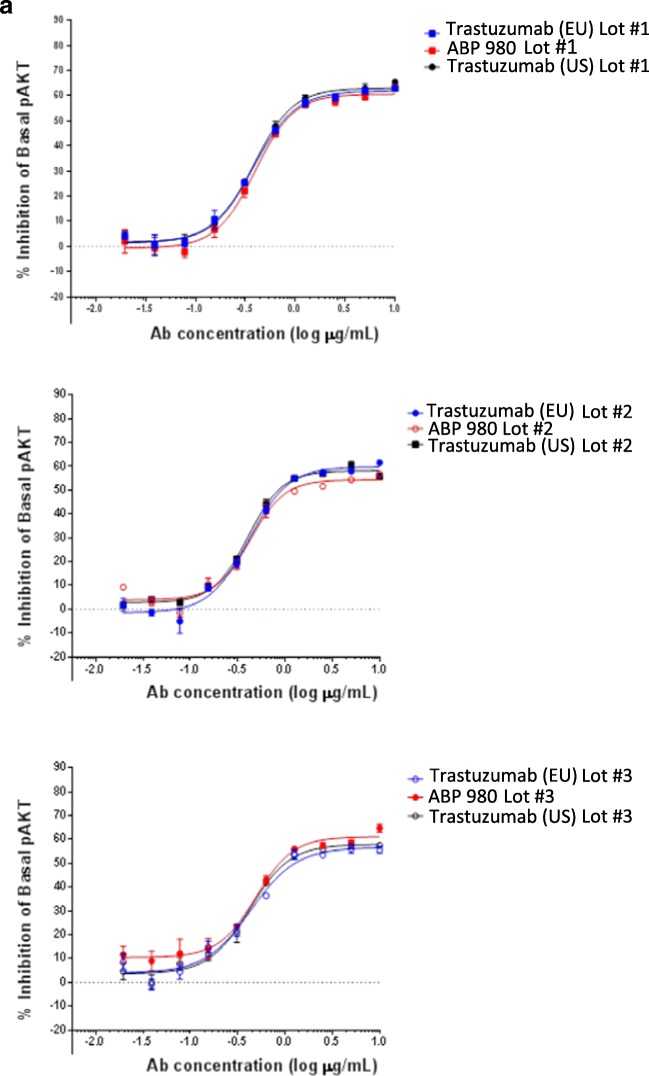

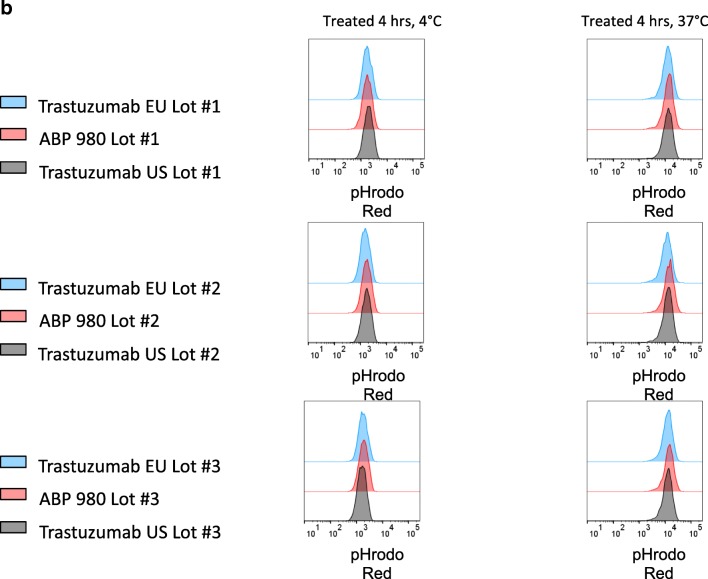

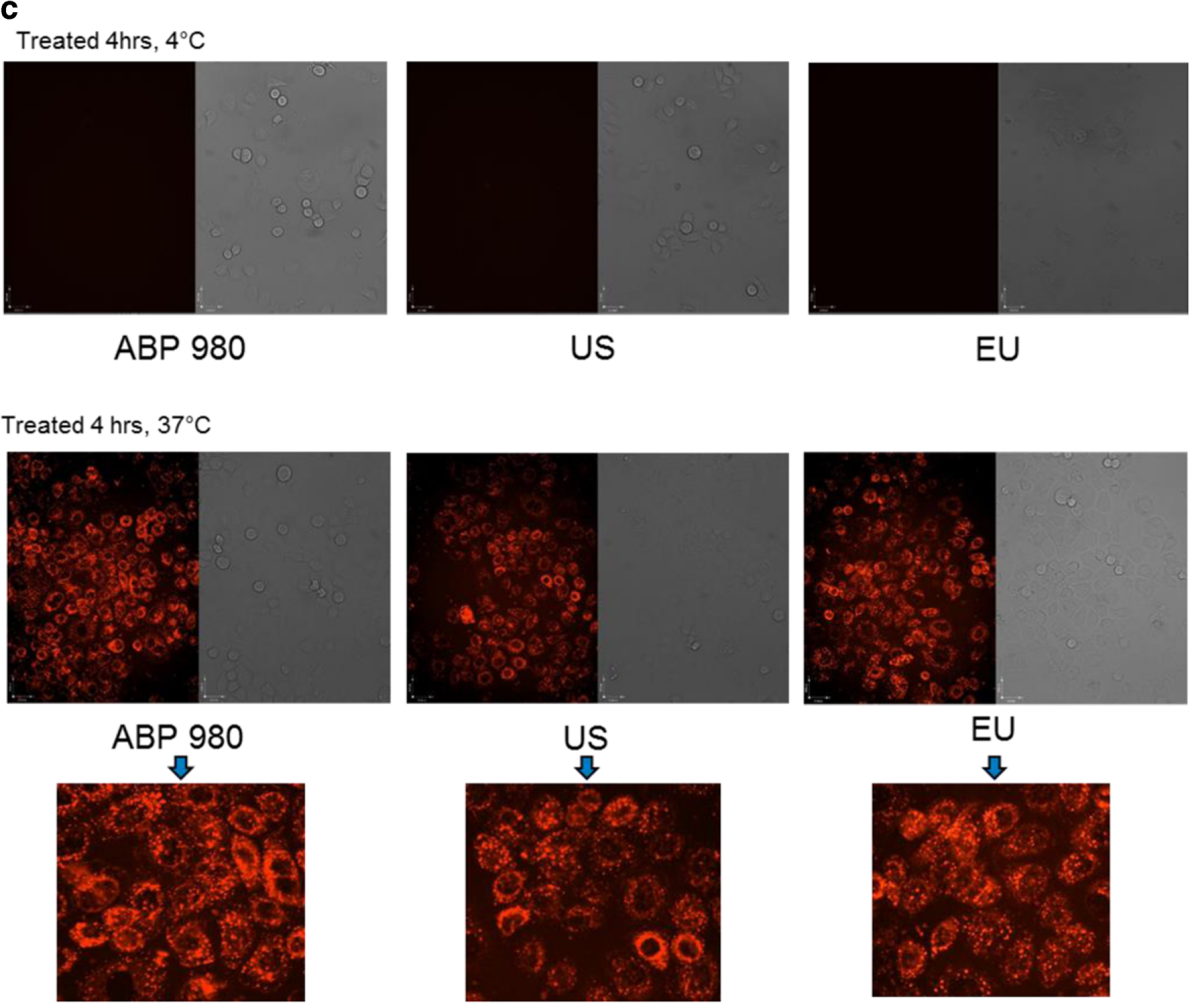
**(a)** ABP 980, trastuzumab (EU) and trastuzumab (US) show similar inhibition of pAKT phosphorylation in breast cancer cells. Graphs show the percent inhibition compared with an untreated control for 3 sets of side-by-side comparisons of unique lots. Each point is a mean of 4 replicates (±SE). Abbreviations: *Ab* antibody, *pAKT* phospho-AKT, *trastuzumab (EU)* EU authorized trastuzumab, *trastuzumab (US)* FDA licensed trastuzumab. **(b)** ABP 980, trastuzumab (EU) and trastuzumab (US) mediate similar HER2 internalization in breast cancer cells. Representative experiment showing fluorescence intensity histogram overlays displaying grouped comparisons between 3 antibody sources and each with 3 respective lots, i.e., EU lot (blue), US lot (grey), and ABP 980 lot (red), at either 4 °C or 37°C. The x axis represents pHrodo™ Red signal fluorescence intensity, and the y-axis represents normalized cell frequency. **(c)** Representative experiment showing single plane confocal micrographs of SK-BR-3 cells treated with pHrodo™ Red-conjugated ABP980, trastuzumab (US) or trastuzumab (EU) at 4°C or 37°C for 4 h. Internalized antibody appears as red fluorescent vesicles, suggesting internalization of the pHrodo Red-conjugated trastuzumab antibodies. Bottom panel images represent digital zooms from their respective original picture. Imaged with a 20×, 0.75 NA objective; excitation with a 561 nm laser, emission filter of 580/20. Abbreviations: *Trastuzumab (EU) or EU* EU authorized trastuzumab, *trastuzumab (US) or US* FDA licensed trastuzumab.

Given that similarity in HER2 kinetic and cell binding was demonstrated, it was also of interest to evaluate the ability of ABP 980 to inhibit constitutively active HER2-induced downstream signaling. Inhibition of HER2-mediated downstream signaling (basal phospho-Akt) by ABP 980, trastuzumab (US), and trastuzumab (EU) was evaluated in BT-474 human breast cancer cells (26). Phospho-Akt (Ser473) was determined using a sandwich immunoassay. The inhibition of basal levels of pAkt in BT-474 cells was compared qualitatively for 3 lots each of ABP 980, trastuzumab (US), and trastuzumab (EU) (Fig. 1a). The overlapping dose response curves support the conclusion that ABP 980 has similar activity as compared with trastuzumab inhibition of downstream signaling mediated by constitutively active HER2.

### HER2 Internalization

Binding of trastuzumab or ABP 980 to HER2 can induce internalization of the receptor, removing the target from the cell surface (4). This activity is a consequence of binding to the HER2 target protein, and the internalization assay provides additional characterization of functional target binding activity of ABP 980. Flow cytometric analysis of HER2-expressing SK-BR-3 cells treated with ABP 980, trastuzumab (EU), and trastuzumab (US) show that these antibodies are readily internalized into cells as shown by the increase in the mean fluorescence intensity of pHrodo™ Red signal in cells treated at 37°C relative to the 4°C controls (Fig. 1b). The level of internalization of ABP 980 is similar to that of the reference product as determined by 3 independent experiments using 3 different lots of fluorescently labeled ABP 980, trastuzumab (EU), and trastuzumab (US). A representative histogram plot analysis of ABP 980 and trastuzumab shows significant overlap, supporting the conclusion that ABP 980 has similar internalization activity as compared with trastuzumab (Fig. 1b).

To confirm that the change in mean fluorescence intensity corresponds to actual internalization of the labeled antibodies, confocal microscopy was performed on cells treated at 37°C. The test samples are internalized, appearing as red patches (puncta) in the cytoplasm of the cells. ABP 980 shows very similar subcellular localization and signal intensity as compared with the trastuzumab lots (Fig. 1c). Note that negative control cells at Time 0 or after 4 h at 4°C showed no signs of internalization, as seen by lack of pHrodo™ Red positive signal, confirming the specificity of internalization by ABP 980 and trastuzumab. Overall, the similar histograms and confocal images support that ABP 980 and trastuzumab mediate similar HER2 internalization.

### Inhibition of Proliferation of NCI-N87 Cells and Specificity for MCF7 Cells

**Fig. 2:**
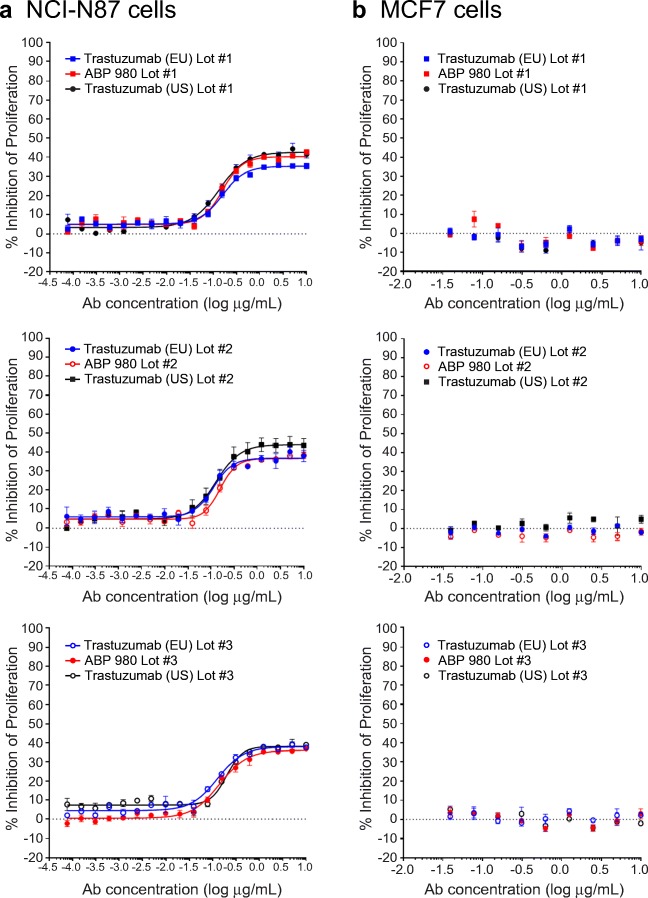
Similarity of ABP 980, trastuzumab (EU) and trastuzumab (US) in the inhibition of proliferation of NCI-N87 cell assay **(a)** and specificity in the MCF7 Assay **(b)**. Graphs show the percent inhibition compared with an untreated control for 3 sets of side-by-side comparisons of unique lots. Each point is a mean of 3 replicates (±SE). Abbreviations: *Ab* antibody, *NCI N87* human gastric cancer cells, *MCF7* non-amplified HER2 breast cancer cells, *trastuzumab (EU)* EU authorized trastuzumab, *trastuzumab (US)* FDA licensed trastuzumab.

Trastuzumab is approved to treat metastatic gastric cancer as well as breast cancer. Thus, an assessment of inhibition of proliferation in gastric cancer cells complements the overall assessment of functional similarity and is relevant to support extrapolation to gastric cancer. Additionally, it is of interest to confirm that specificity for HER2 over-expression is maintained by ABP 980. Thus, 2 cell lines were evaluated for proliferation inhibition: the NCI-N87 gastric cancer cell line, expressing high HER2 levels, and the MCF7 breast cancer cell line, expressing lower levels of HER2 (27). Three lots of ABP 980 and 3 lots each of trastuzumab (US) and trastuzumab (EU) were compared in the 2 cell lines (Fig. 2). The dose response curves for the inhibition of proliferation in the NCI-N87 gastric cancer cell line overlap, supporting the conclusion of similarity of ABP 980 and trastuzumab. Additionally, all of the lots tested showed no inhibition of proliferation in the MCF7 breast cancer cell line, supporting that specificity for HER2 over-expression is maintained by ABP 980.

### Synergy Study

**Fig. 3:**
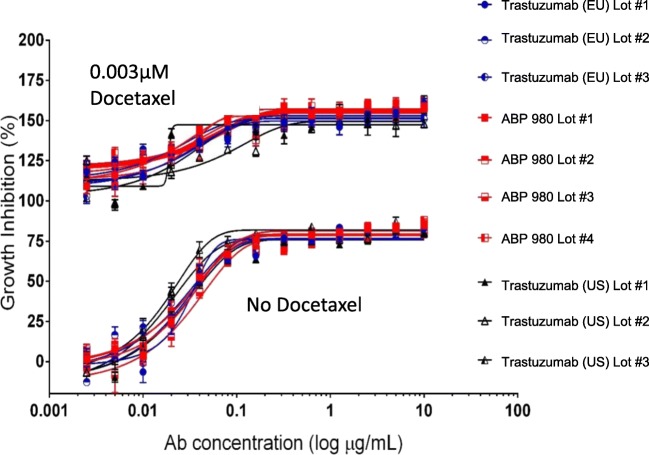
Comparison of docetaxel in combination with trastuzumab (EU), ABP 980, and trastuzumab (US) on the growth of NCI N87 Cells. Graph shows the percent growth inhibition for representative test samples in the presence and absence of docetaxel as compared with an untreated control. Unique lots are shown for ABP 980 (red), trastuzumab (US) (black), and trastuzumab (EU) (blue). Each point is a mean of 6 replicates (±SE). Abbreviations: *trastuzumab (EU)* EU authorized trastuzumab, *trastuzumab (US)* FDA licensed trastuzumab.

Trastuzumab enhances the activity of multiple chemotherapeutics and is approved for use in the clinic in combination with standard of care chemotherapy for breast and gastric cancer (2). Docetaxel is a taxane chemotherapy that synergizes with trastuzumab in clinical use (28,29) as well as in *in vitro* synergy studies (30). Thus, it was of interest to evaluate the similarity of ABP 980 and trastuzumab in a setting where synergy with a chemotherapeutic could be evaluated. After method optimization in NCI-N87 gastric cancer cells to determine concentrations of ABP 980 and docetaxel where synergy could be evaluated, the CombinatoRx™ method was used to examine the synergistic inhibition of proliferation of NCI-N87 cells for 4 lots of ABP 980 and 3 lots each of trastuzumab (US) and trastuzumab (EU). Similar synergy scores were obtained for activity with docetaxel across all lots of trastuzumab and ABP 980 in NCI-N87 cells (Supplemental Table I). Visualization of representative combination activity from extracted dose response curves at 0.003 μM docetaxel compared with ABP 980 alone demonstrates that the curves overlap and are aligned with similar synergy scores (Fig. 3). Overall, the synergy results support the conclusion of similarity of ABP 980 and trastuzumab in the presence of docetaxel chemotherapeutic.

### FcγR Binding as Assessed by SPR

ABP 980 and trastuzumab mediate effector functions (antibody-dependent cell-mediated cytotoxicity [ADCC] and antibody-dependent cellular phagocytosis [ADCP]) via their Fc binding domains. A thorough characterization of the relative binding activities for FcγRIIa (131H) and FcγRIIIa have been reported, demonstrating the similar Fc-mediated binding properties of ABP 980 (16). Here, we show the binding results using SPR comparing the binding activity of 4 lots of ABP 980 and 3 lots each of trastuzumab (US) and trastuzumab (EU) for the following: FcγRs (FcγRIa, FcγRIIa (131H), FcγRIIb, FcγRIIIa (158 V), FcγRIIIa (158F), and FcγRIIIb). This analysis complements the relative binding results previously presented in addition to providing a binding comparison for additional FcγRs where no clinical role has been attributed to binding activity. It is important to note that multiple orthogonal methods, including associated effector functions and binding to FcγR on expressing primary cells, are employed to evaluate the similarity of FcγR binding because no one method provides all of the necessary information to thoroughly evaluate whether minor analytical differences will have a meaningful impact on binding *in vivo*.

**Fig. 4:**
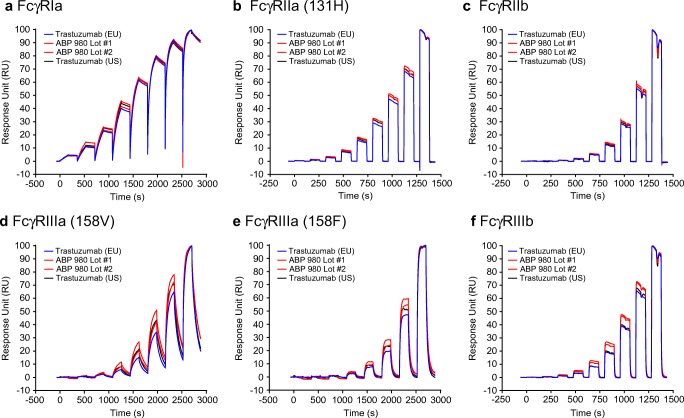
Similar binding of ABP 980, trastuzumab (EU) and trastuzumab (US) to Fcγ receptors. Representative sensorgram overlays are shown for 2 lots of ABP 980 (red) and a single lot each of trastuzumab (US) (black) and trastuzumab (EU) (blue). **(a)** FcγRIa **(b)** FcγRIIa (131H) **(c)** FcγRIIb **(d)** FcγRIIIa (158 V) **(e)** FcγRIIIa (158F) **(f)**. FcγRIIIb. Abbreviations: *trastuzumab (EU)* EU authorized trastuzumab, *trastuzumab (US)* FDA licensed trastuzumab.

FcγR binding analysis in this study was performed by injecting a series of concentrations of ABP 980, trastuzumab (US), or trastuzumab (EU) over captured FcγRs using a Biacore T200. To directly compare the sensorgram similarity of each sample, normalized average sensorgrams from the data (*n* = 3 replicates) were generated for each sample and overlaid. Representative results are shown from 1 lot each of trastuzumab (US) and trastuzumab (EU) as compared with 2 lots of ABP 980 (Fig. 4). Additionally, the equilibrium dissociation constants (K_d_) from the displayed sensorgrams are reported in Table III. The sensorgram overlays for all FcγRs tested are comparable with some minor variability (10%–20%) noted in the binding response during the association phase of FcγRIIIa and FcγRIIIb, with no impact observed on the overall kinetic profile of ABP 980 and trastuzumab or the reported relative affinities. Taken with the other assessments of FcγR binding and function, the kinetic binding results support the conclusion that ABP 980 has similar Fc-mediated binding activity as compared with trastuzumab.

**Table III:** Equilibrium Dissociation Constants (K_d_) of ABP 980, Trastuzumab (EU) and Trastuzumab (US) to FcγRIa, FcγRIIa (131H), FcγRIIb, FcγRIIIa (158 V), FcγRIIIa (158F), and FcγRIIIb

Sample	FcγRIa (pM)	FcγRIIa (131H) (μM)	FcγRIIb (μM)	FcγRIIIa (158 V) (nM)	FcγRIIIa (158F) (nM)	FcγRIIIb (μM)
Trastuzumab (EU) Lot #1	536	8.36	31.0	193	822	13.8
ABP 980 Lot #1	468	6.91	26.3	105	478	8.14
ABP 980 Lot #2	405	5.52	22.0	91.3	463	8.29
Trastuzumab (US) Lot #1	405	5.74	24.6	146	707	11.8

*K*_*d*_ *(pM) is reported in the table above; Trastuzumab (EU)* EU authorized trastuzumab, *trastuzumab (US)* FDA licensed trastuzumab

### Binding to Primary NK Cells and Primary Monocytes

To complement the SPR results, binding to primary effector cells, where multiple FcγRs may be expressed, was also assessed to provide an integrated measure of binding similarity of ABP 980 as compared with trastuzumab. Peripheral blood mononuclear cells (PBMCs) from 1 donor with a (131 R/R; 158 F/F) genotype and 1 donor with a (131 H/R; 158 V/V) genotype were used for comparative testing. A competitive binding assay was used, in which test article was allowed to compete with fluorescently labeled reference standard for binding on PBMCs. Histogram plots were prepared and overlaid for comparison.

**Fig. 5:**
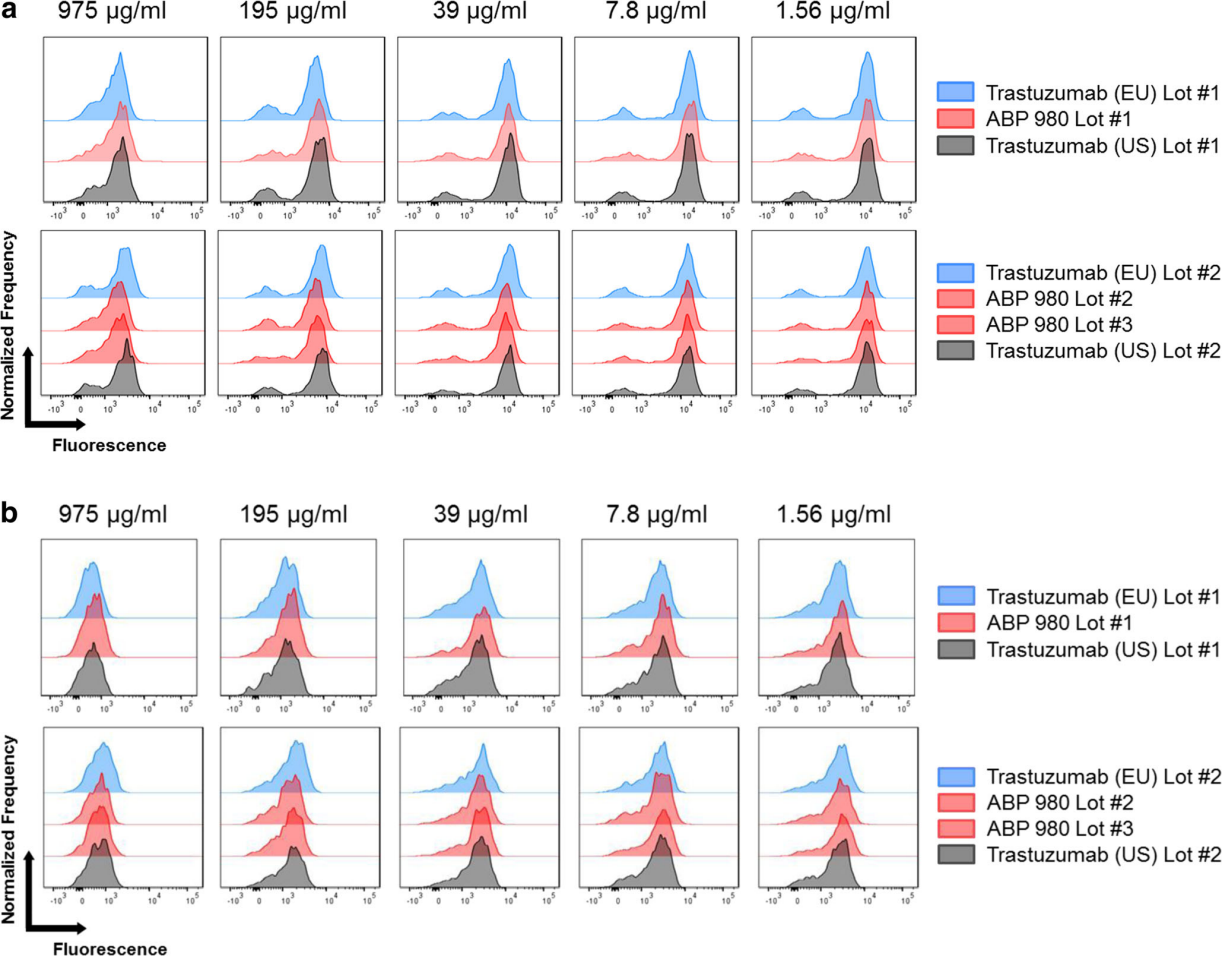
Similar primary NK cell binding by ABP 980, trastuzumab (EU) and trastuzumab (US). Lots are grouped from the same assay plate for side-by-side comparison using histogram overlays from select concentrations in the dose matrix. X-axis represents fluorescently labeled reference standard where higher binding is indicated by increased fluorescence. Y-axis represents normalized frequency. **(a)** Results from a homozygous donor for the high affinity variant at position 158 in FcγRIIIa **(b)** Results from a homozygous donor for the low affinity variant at position 158 in FcγRIIIa. Abbreviations: *trastuzumab (EU)* EU authorized trastuzumab, *trastuzumab (US)* FDA licensed trastuzumab.

Natural killer (NK) cells primarily express FcγRIIIa, the FcγR primarily responsible for mediating ADCC. FcγRIIIa has 2 common allelic variants at position 158 that have been characterized as high affinity (158 V [Valine]) and low affinity (158F [Phenylalanine]). Binding to primary NK cells was evaluated as an orthogonal method to bridge the relative FcγRIIIa binding assays, and the PBMC ADCC assay which was used to evaluate similarity (16). Three lots of ABP 980, 2 lots of trastuzumab (US), and 3 lots of trastuzumab (EU) were compared. Representative overlays show that the histograms overlap significantly for both donors (Fig. 5). This is consistent with the conclusion that ABP 980 has similar FcγRIIIa binding on primary NK cells as compared with trastuzumab.

**Fig. 6:**
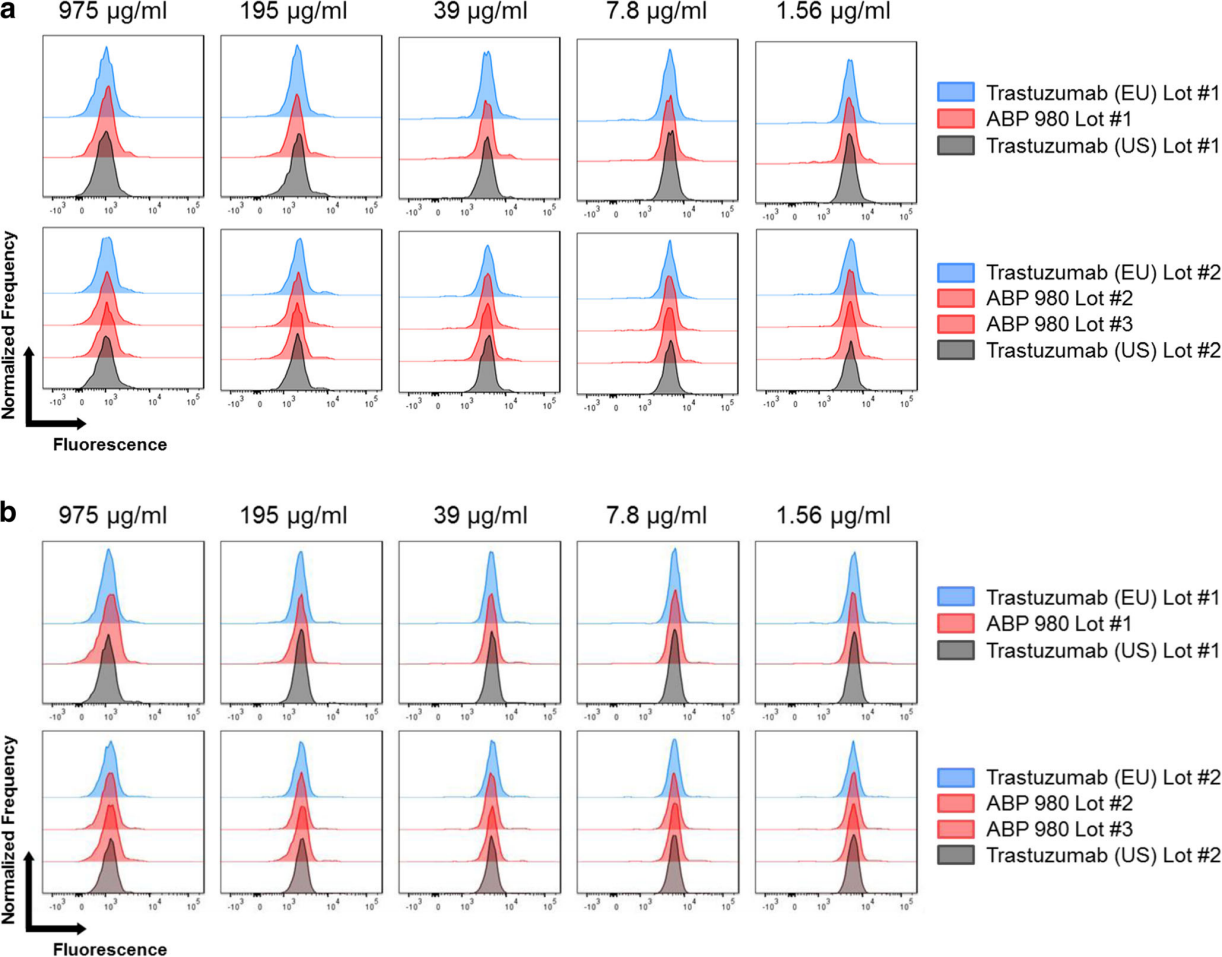
Similar primary monocyte cell binding by ABP 980, trastuzumab (EU) and trastuzumab (US). Lots are grouped from the same assay plate for side-by-side comparison using histogram overlays from select concentrations in the dose matrix. X-axis represents fluorescently labeled reference standard where higher binding is indicated by increased fluorescence. Y-axis represents normalized frequency. **(a)** Results from a homozygous donor for the arginine variant at position 131 in FcγRIIa **(b)** Results from a heterozygous donor for position 131 FcγRIIa. Abbreviations: *trastuzumab (EU)* EU authorized trastuzumab, *trastuzumab (US)* FDA licensed trastuzumab.

Monocytes express multiple Fcγ receptors, including FcγRIa, FcγRIIa, and FcγRIIb. FcγRIa is the high-affinity receptor for IgG1 antibodies, and a specific role for this receptor in the safety or efficacy of trastuzumab has not been identified. FcγRIIa is the primary FcγR responsible for mediating ADCP (31,32). FcγRIIb can modulate ADCP activity through its inhibitory function. Binding to primary monocytes was evaluated as an orthogonal method to bridge the relative FcγRIIa (131H) binding assays and the PBMC ADCP assay. FcγRIIa has 2 common allelic variants at position 131 that have been characterized as high (131H) and low (131R) responder forms (33). One donor with a (131 R/R) genotype and 1 donor with a (131 H/R) genotype were used to perform testing to complement the relative binding study performed with the 131 H variant (16). The histograms overlap significantly for all lots tested in both donors, consistent with the conclusion that ABP 980 and trastuzumab have similar FcγR binding on primary monocytes (Fig. 6).

### ADCP

**Fig. 7:**
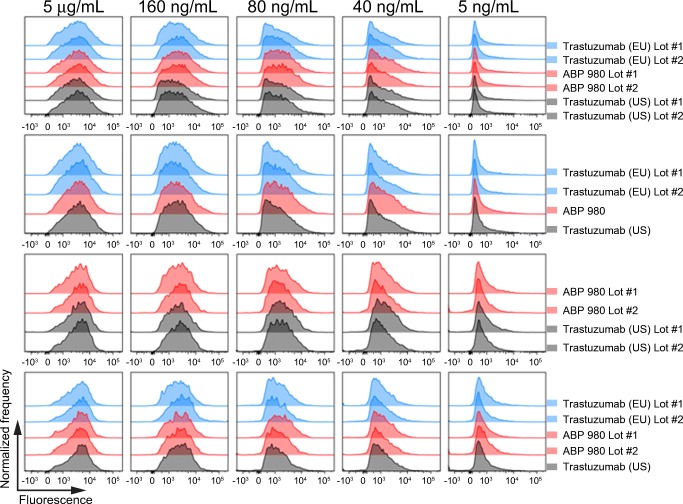
Similar ADCP activity of ABP 980, trastuzumab (EU) and trastuzumab (US). Lots are grouped from the same assay plate for side-by-side comparison using histogram overlays from select concentrations in the dose matrix. Abbreviations: *trastuzumab (EU)* EU authorized trastuzumab, *trastuzumab (US)* FDA licensed trastuzumab.

ADCP activity has been reported in trastuzumab-treated patient PBMCs (34). Human PBMCs were incubated with carboxyfluorescein succinimidyl ester (CFSE)-labeled SK-BR-3 breast cancer cells and after allowing time for ADCP to occur, cells were subsequently stained to mark monocytes (CD14) and evaluate viability (propidium iodide). After washing, live CD14+ cells were acquired by flow cytometry. The portion of live CD14+ cells that were positive for CFSE fluorescence was considered to be a direct measure of ADCP activity. Histograms representing the number of events and fluorescence intensity were plotted and overlaid for 7 lots of ABP 980, 6 lots of trastuzumab (US), and 6 lots of trastuzumab (EU) in 4 side-by-side experiments (Fig. 7). The extensive overlap in the histograms shows that ABP 980 and trastuzumab have similar phagocytic activity against breast cancer cells.

### *In Vivo* Pharmacology and Toxicokinetics

Two xenograft tumor models overexpressing HER2, BT-474 (human breast cancer cells), and NCI-N87 (human gastric cancer cells), were used to compare the effects of ABP 980 and trastuzumab to demonstrate that ABP 980 has a similar ability to inhibit tumor growth *in vivo* as well as *in vitro*.

**Fig. 8:**
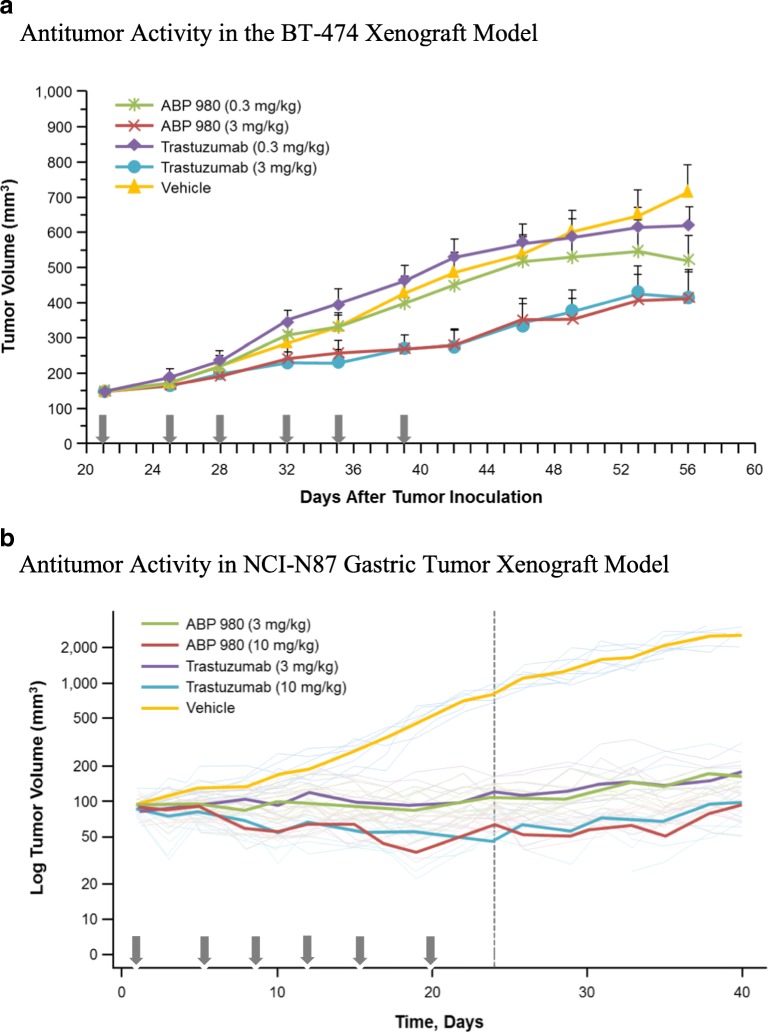

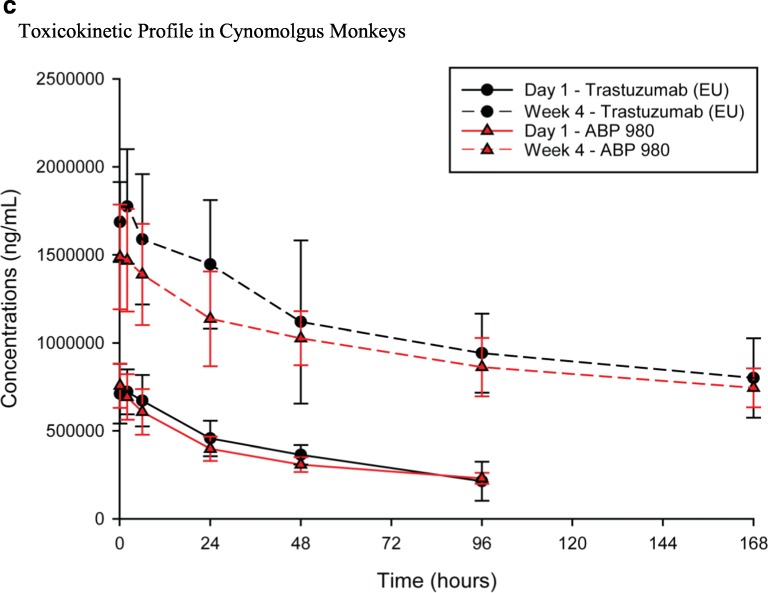
Non-clinical pharmacology and toxicokinetic results for ABP 980 and trastuzumab (EU). **(a)** Antitumor activity in the BT-474 xenograft model. Arrows indicate dosing days. Data are represented as mean ± standard error of the mean for each group (*N* = 10 mice/group). BT-474 = metastatic human breast cancer cells. **(b)** Antitumor activity in the NCI-N87 xenograft model. Arrows indicate dosing days. Dark lines represent mean tumor volume (N = 10 mice/group); dotted lines represent Day 22, the end of the treatment period. NCI-N87 = human gastric cancer cells. **(c)** Toxicokinetic profiles (mean ± SD) for test articles in cynomolgus monkey. Abbreviations: *trastuzumab (EU)* EU authorized trastuzumab.

Non-obese diabetic/severe combined immunodeficiency (NOD/SCID) mice were injected orthotopically with BT-474 tumor cells at a concentration of 8 × 10^6^ per mouse. Doses were chosen based on a previously published study using trastuzumab (35). Mice were implanted subcutaneously (SC) with estrogen pellets to support the growth of BT-474 cells, which are estrogen receptor positive. Twenty-one days later, when the average tumor size reached approximately 146 mm^3^, treatment with vehicle, ABP 980 (0.3 mg/kg or 3.0 mg/kg), or trastuzumab (EU) (0.3 mg/kg or 3.0 mg/kg) was administered twice weekly intravenously (IV) for 19 days. Tumor sizes were measured twice per week until Day 56. Statistical analyses of the difference in tumor volume among the groups were conducted with the data obtained at Day 42 post-tumor inoculation (Fig. 8a). The low-dose groups of ABP 980 and trastuzumab (0.3 mg/kg) showed no significant antitumor activity compared with the vehicle control group. Inhibition of tumor growth was similar between ABP 980 and trastuzumab at 3 mg/kg, and tumor growth inhibition by ABP 980 and trastuzumab was significantly greater than the vehicle control group. Mean tumor size in the vehicle control group was 487 mm^3^ on Day 42 and 284 mm^3^ in the ABP 980 3 mg/kg group, whereas the mean tumor size in the trastuzumab 3 mg/kg group was 280 mm^3^ at Day 42 post-tumor inoculation. At Day 42 post-inoculation, both ABP 980 and trastuzumab given at 3 mg/kg significantly inhibited tumor growth compared with the vehicle control (*p* = 0.013 and *p* = 0.011, respectively), supporting the overall conclusion of similarity of ABP 980 (Supplemental Table II).

A second tumor model (NCI-N87) was used to compare the antitumor activity of ABP 980 and trastuzumab in gastric cancer cells. ABP 980 and trastuzumab doses were chosen based on previously published studies in NCI-N87 xenograft models using trastuzumab (36,37). Briefly, immunodeficient female CD-1 nude mice were injected SC with NCI-N87 tumor cells at a concentration of 5 × 10^6^ cells per mouse. When the average tumor size was approximately 100 mm^3^, vehicle control, ABP 980 (3 mg/kg or 10 mg/kg) or trastuzumab (EU) (3 mg/kg or 10 mg/kg) was administered twice weekly by intraperitoneal injection for 19 days. Tumors were allowed to regrow after dosing was discontinued, and tumor sizes were measured 3 times per week until Day 35. Tumor volume is presented using a ratio-to-baseline adjusted area under the tumor volume curve (AUCr). The AUCr was used in the calculation of treatment/control (T/C) ratios (tumor volume in treatment group/tumor volume in comparator group) to capture the full change in tumor size over the treatment period, and statistical analysis was performed using those results (Fig. 8a–b). ABP 980 and trastuzumab were similarly effective in inhibiting tumor growth at 3 and 10 mg/kg (Fig. 8b). All treatment groups were significantly different compared with vehicle control based on the T/C ratio (Supplemental Table III). Similarity in tumor growth inhibition between ABP 980 and trastuzumab was tested by calculating the T/C ratios and 2-sided 90% confidence limits. Tumor growth inhibition was similar between ABP 980 and trastuzumab (T/C ratios for ABP 980/trastuzumab were <100% and the upper confidence limits were >100) (Supplemental Table III). Together, the pharmacology studies conducted in breast and gastric cancer xenograft models support the conclusion that ABP 980 is biosimilar to trastuzumab.

The multiple-dose toxicokinetics (TK) of ABP 980 and trastuzumab (EU) in cynomolgus monkeys was evaluated in a good laboratory practice (GLP) toxicology study in which 18 female monkeys were assigned to 1 of 3 treatment groups (*n* = 6/group) receiving vehicle control, ABP 980 25 mg/kg, or trastuzumab (EU) 25 mg/kg IV twice weekly for 4 weeks. Serum samples were collected from all animals for TK analysis on Day 1 at pre-dose, 0.083 (5 min), 2, 6, 24, 48, and 96 h post-dose and on the last day of dosing (Day 22) at pre-dose, 0.083 (5 min), 2, 6, 24, 48, 96, and 168 h post-dose. Serum samples were analyzed for ABP 980 and trastuzumab concentrations using a validated ELISA method with a lower limit of quantification of 200 ng/mL. Individual and mean serum concentrations were measured after administration of ABP 980 or trastuzumab, and TK parameters were calculated to compare the TK of ABP 980 and trastuzumab. The mean (± standard deviation) serum concentration-time profiles are presented (Fig. 8c). In addition, the TK parameter results are also summarized (Supplemental Table IV). Following ABP 980 and trastuzumab IV bolus injections, mean area under the serum drug concentration-time curve (AUC) from time 0 to 96 h post dose (AUC_0-96h_) values were similar at 34,501 μg•hr./mL for ABP 980 and 37,215 μg•hr./mL for trastuzumab (EU) after dosing on Day 1 and 102,685 μg•hr./mL and 117,721 μg•hr./mL, respectively, in Week 4, on Day 22; therefore, ABP 980 and trastuzumab exposure was determined to be similar, supporting the overall conclusion of biosimilarity.

## Discussion

The *in vitro* and *in vivo* pharmacologic assessment of similarity is intended to comprehensively compare the activity of ABP 980 and trastuzumab in functions known to be important to the mechanisms of action (MOAs) of trastuzumab. The similarity of ABP 980 has been assessed using a comprehensive analytical and functional studies (16). This work complements the primary assessment with additional *in vitro* functional assays, binding studies, and non-clinical studies and informs the TOE with respect to *in vitro* and *in vivo* functional similarity of ABP 980 compared with trastuzumab. These results also support the scientific justification of extrapolation to all approved conditions of use given the established functional similarity of the 2 products and the same MOA in all conditions of use.

The primary MOA requires binding to the extracellular domain of HER2 (4). Binding kinetics to the HER2 target as well as relative cell binding to HER2 over-expressing breast cancer cells were shown to be similar. Additionally, HER2 internalization and downstream signaling through PI3K-AKT, the key mediator of HER2 ligand-independent cell proliferation was also similar. Inhibition of proliferation was shown to be similar in NCI-N87 gastric cancer cells and specific for HER2 over-expression, supporting potency results from the comprehensive similarity assessment and representing a cell type from an extrapolated indication.

Trastuzumab has been used clinically in combination with chemotherapeutic agents, including docetaxel. Using a synergy combination matrix approach, ABP 980 was shown to have similar synergy with docetaxel in the growth inhibition of NCI-N87 gastric cancer cells, supporting overall similarity and extrapolation to gastric cancer.

Multiple assays were used to characterize the Fc portion of ABP 980 as compared with trastuzumab. In addition to similar FcγRIIa (131H) and FcγRIIIa relative binding presented in Hutterer and colleagues, sensorgram overlays were compared for the additional FcγRs (FcγRIa, FcγRIIb, and FcγRIIIb) as well as to a side-by-side comparison of FcγRIIa (131H), FcγRIIIa (158 V) and FcγRIIIa (158F) binding (16). Primary cell binding was also evaluated to confirm the similarity of ABP 980 as a bridge between the relative binding FcγR assays and primary cell functional assays (ADCP) as primary cells provide an integrated assessment of binding where expression of multiple FcγRs occurs naturally. Binding to primary NK cells from healthy genotyped donors homozygous for either the high (158 V) or low (158F) variant of FcγRIIIa was shown to be similar based on competitive cell binding. Additionally, binding to primary monocytes from healthy genotyped donors heterozygous for position 131 in FcγRIIa or homozygous for arginine at that position was also shown to be similar, supporting the overall similarity of ABP 980 to trastuzumab.

ADCP has been detected in trastuzumab-treated patient PBMCs (34) and in isolated monocytes (38), although the relative contribution of this effector function to the clinical efficacy or safety of trastuzumab has not been established. Using a PBMC-based ADCP method, ABP 980 and trastuzumab were shown to be similar for phagocytic activity against breast cancer cells.

An *in vivo* assessment in 2 xenograft tumor models over-expressing HER2, BT-474 and NCI-N87, was used to demonstrate that, like trastuzumab, ABP 980 has the ability to inhibit tumor growth in both breast and gastric cancer tumors in an *in vivo* setting. Results from these xenograft models show that ABP 980 and trastuzumab are similarly able to inhibit tumor growth. Furthermore, the results suggest that ABP 980 inhibits tumor growth in a manner that is consistent with the known MOA of trastuzumab across indications, supporting extrapolation.

The TK assessment in a multiple-dose toxicology cynomolgus monkey study showed a similar *in vivo* exposure between the ABP 980 and trastuzumab treatment groups following a single dose on Day 1 as well as after multiple dosing at Week 4. The approximately 3-fold dose accumulation observed after multiple dosing was also similar between the treatment groups.

## Conclusion

In summary, the results presented here contribute to the TOE with respect to functional similarity of ABP 980 to trastuzumab, supplementing the previous report of analytical and functional similarity with additional assays for key functions of the molecules including *in vitro* binding (HER2 relative cell binding and binding kinetics, FcγR cell and kinetic binding), additional aspects of effector and primary HER2 inhibition (ADCP, inhibition of HER2 signaling, inhibition of proliferation in gastric cancer cells, synergy with chemotherapeutic and HER2 internalization) as well as non-clinical pharmacology (tumor xenograft studies in breast and gastric cancer models) and toxicokinetic results. These results further limit residual uncertainty regarding the previously reported minor analytical differences (16) and provide additional confidence in the similarity of ABP 980 and trastuzumab for all functional aspects of the molecules *in vitro* and *in vivo*. Additionally, these results also support the scientific justification of extrapolation to all approved conditions of trastuzumab given the established functional similarity of the products and the conserved mechanisms of action across all conditions of use.

## Electronic supplementary material

ESM 1 (DOCX 29 kb)
