# Supplementary material for: Functional and Nonclinical Similarity of ABP 980, a Biosimilar of Trastuzumab
Source: Pharm Res. 2019 Nov 6;36(12):177. doi: 10.1007/s11095-019-2702-8 (PMC6834741; doi:10.1007/s11095-019-2702-8)
Supplement: Supplementary file 1 — (DOCX 29 kb) [file 11095_2019_2702_MOESM1_ESM.docx]

**Supplemental Tables**

**Supplemental Table I**  Synergy Scores Comparing ABP 980, Trastuzumab (EU) and Trastuzumab (US) in Combination with Docetaxel in Gastric Cancer Cells

| Plus Docetaxel (NCI-N87) | Synergy Score (Mean ± SD) |
| --- | --- |
| Trastuzumab (EU) | 15.8 ± 1.3 |
| ABP 980 | 16.1 ± 1.4 |
| Trastuzumab (US) | 16.4 ± 1.8 |

Synergy scores are shown using the mean ± standard deviation (SD) for 4 lots of ABP 980 and 3 lots each of trastuzumab sourced from the US or EU.

*Trastuzumab (EU)* EU authorized trastuzumab, *trastuzumab (US)* FDA licensed trastuzumab

**Supplemental Table II**  Comparison of ABP 980 and Trastuzumab Anti-tumor Activity in the BT-474 Xenograft Model

| Treatment | Compared with | Dose (mg/kg) | T/C | p-value (Tumor Size) |
| --- | --- | --- | --- | --- |
| Trastuzumab (EU) | Vehicle | 0.3 | 109 | 0.89 |
| Trastuzumab (EU) | Vehicle | 3 | 53 | 0.011* |
| ABP 980 | Vehicle | 0.3 | 93 | 0.97 |
| ABP 980 | Vehicle | 3 | 58 | 0.013* |

*Trastuzumab (EU)* EU authorized trastuzumab, *trastuzumab (US)* FDA licensed trastuzumab, *T/C* ratio of tumor volume for a treatment (ABP 980 or trastuzumab)/tumor volume for the control (vehicle) at Day 42. Tumor volume was calculated using a ratio-to-baseline adjusted AUCr –. Statistical significance is indicated as * p </= 0.05

**Supplemental Table III**  Comparison of ABP 980 and Trastuzumab Anti-tumor Activity in the NCI-N87 Xenograft Model

| Treatment | Compared with | Dose (mg/kg) | T/C | Upper 95% Confidence Limit |
| --- | --- | --- | --- | --- |
| Trastuzumab (EU) | Vehicle | 3 | 40 | 58.7 |
| Trastuzumab (EU) | Vehicle | 10 | 27 | 31.6 |
| ABP 980 | Vehicle | 3 | 36 | 42.5 |
| ABP 980 | Vehicle | 10 | 25 | 32.2 |

*Trastuzumab (EU)* EU authorized trastuzumab, *trastuzumab (US)* FDA licensed trastuzumab, *T/C* ratio of tumor volume for a treatment (ABP 980 or trastuzumab)/tumor volume for the control (vehicle). Tumor volume was calculated using a ratio-to-baseline adjusted AUCr –

**Supplemental Table IV**  Summary of Toxicokinetic Parameters after Single (Day 1) or Repeated Twice Weekly (Week 4) Intravenous Injections of Trastuzumab or ABP 980 to Female Cynomolgus Monkeys.

| Period | Parameters | Units | Trastuzumab  (EU) | ABP 980 | Ratio Trastuzumab/  ABP 980 |
| --- | --- | --- | --- | --- | --- |
| Day 1 | tmax* | h | 2 | 0.0833 | na |
|  | Cmax | μg/mL | 814 | 773 | 1.05 |
|  | C0 | μg/mL | 713 | 760 | 0.94 |
|  | AUC0-96h | μg.h/mL | 37215 | 34501 | 1.08 |
|  | t1/2 | h | 95.8 | 93.3 | 1.03 |
|  | AUC0-inf | μg.h/mL | 75912 | 65895 | 1.15 |
| Week 4 | tmax* | h | 4 | 1.0417 | na |
|  | Cmax | μg/mL | 1939 | 1722 | 1.13 |
|  | C0 | μg/mL | 1690 | 1501 | 1.13 |
|  | AUC0-96h | μg.h/mL | 117721 | 102685 | 1.15 |
|  | AUC0-168h | μg.h/mL | 180435 | 160535 | 1.12 |
|  | t1/2 | h | 193 | 178 | 1.08 |
|  | AUC0-inf | μg.h/mL | 399175 | 355745 | 1.12 |
| AUC0-96h Week 4 / AUC0-96h Day 1 | | | 3.24 | 3.01 | na |
| AUC0-96h Week 4 / AUC0-inf Day 1 | | | 1.96 | 1.61 | na |

*median was calculated instead of mean.

*na* not applicable, *C0* concentration at t = 0 h, used for the AUC calculation
